# Coccidioidomycosis and Host Microbiome Interactions: What We Know and What We Can Infer from Other Respiratory Infections

**DOI:** 10.3390/jof9050586

**Published:** 2023-05-18

**Authors:** Susana Tejeda-Garibay, Katrina K. Hoyer

**Affiliations:** 1 Quantitative and Systems Biology, Graduate Program, University of California Merced, Merced, CA 95343, USA; 2 Department of Molecular and Cell Biology, University California Merced, Merced, CA 95343, USA; 3 Health Sciences Research Institute, University of California Merced, Merced, CA 95343, USA

**Keywords:** Valley fever, coccidioidomycosis, *Coccidioides*, microbiota, antibiotic treatment, infection, immune response

## Abstract

Between 70 and 80% of Valley fever patients receive one or more rounds of antibiotic treatment prior to accurate diagnosis with coccidioidomycosis. Antibiotic treatment and infection (bacterial, viral, fungal, parasitic) often have negative implications on host microbial dysbiosis, immunological responses, and disease outcome. These perturbations have focused on the impact of gut dysbiosis on pulmonary disease instead of the implications of direct lung dysbiosis. However, recent work highlights a need to establish the direct effects of the lung microbiota on infection outcome. Cystic fibrosis, chronic obstructive pulmonary disease, COVID-19, and *M. tuberculosis* studies suggest that surveying the lung microbiota composition can serve as a predictive factor of disease severity and could inform treatment options. In addition to traditional treatment options, probiotics can reverse perturbation-induced repercussions on disease outcomes. The purpose of this review is to speculate on the effects perturbations of the host microbiome can have on coccidioidomycosis progression. To do this, parallels are drawn to aa compilation of other host microbiome infection studies.

## 1. Coccidioides Overview

*Coccidioides posadasii* and *Coccidioides immitis* are the fungal pathogens responsible for the pulmonary disease coccidioidomycosis, also known as Valley fever. Disease is contracted upon inhalation of *Coccidioides* fungal spores following soil disruption caused by high winds or digging. *Coccidioides* is currently endemic to hot and dry regions of the Western and Southwestern United States, Central America, and South America [1]. These thread-like mycelia grow and divide during periods of rainfall and desiccate into spore-containing fragments during dry periods. During dry periods, the infectious arthroconidia form of the fungus is more likely to become aerosolized and inhaled, becoming a threat to field and construction workers. As many as 60% of Valley fever cases remain asymptomatic, 40% experience flu-like symptoms that resolve on their own, and 5–10% of symptomatic patients develop chronic pulmonary or disseminated disease [1]. These statistics, however, do not likely represent the true number of cases as many physicians are not well-versed in diagnosing the disease because of its region specificity. coccidiodomycosis is also often misdiagnosed as bacterial pneumonia. Inaccurate diagnosis further results in about 80% of patients being treated with several rounds of antibiotics prior to accurate diagnosis [2]. In addition to antibiotic resistance, antibiotic treatment has downstream implications on host bacterial dysbiosis which have yet to be considered for *Coccidioides* infection. Although disease is endemic to only a few regions, a climate niche model predicts that the area of climate-limited endemicity will more than double, resulting in a 50% increase in Valley fever cases by 2100 as temperatures increase and precipitation patterns change [3]. As the endemic area increases, it is crucial to understand the implication that altering the microbiome through inappropriate antibiotic treatment has on disease outcome [3].

## 2. Respiratory Tract Microbiome

The 2007 Human Microbiome Project characterizes the microbiomes of healthy individuals at five major body sites using 16S and metagenomic shotgun sequencing [4]. This project did not initially include the lungs as a site of investigation because it was long thought to be sterile [4]. Culture-dependent techniques pose a challenge in lung microbiome collection due to low microbial abundance compared to other body sites, in addition to only 1% of lung bacteria being culturable in the laboratory [4,5]. Sequencing methods used to identify microbial communities in the lung reveal that a healthy lung is comprised primarily of the families *Firmicutes*, *Bacteroidetes*, *Proteobacteria*, *Fusobacteria*, and *Actinobacteria* [4,6]. At the operational taxonomic unit level, *Prevotella*, *Veillonella*, and *Streptococcus* are routinely identified as prevalent lung residents [6]. The upper respiratory tract consists of the nose, mouth, sinuses, pharynx, and larynx. The lung is part of the lower respiratory system along with the trachea and primary bronchi. Among healthy individuals, the microbiome of upper and lower respiratory tract is indistinguishable [7]. However, microbiota differ between the upper and lower respiratory tract and even within the lung among individuals with asthma, chronic obstructive pulmonary disease (COPD), and cystic fibrosis [5,8,9,10]. Changes in microbial composition have also been identified as a result of infection and antibiotic treatment. Recent studies on COVID-19 and respiratory syncytial virus (RSV) have identified differences between both respiratory and intestinal microbiomes during viral infection and due to antibiotic treatment [11,12,13,14].

## 3. Microbiome Changes during Perturbation

Changes in the host microbiome can occur due to a myriad of perturbations including infection, antibiotic treatment, or infection accompanied by antibiotic treatment. These perturbations can result in proximal or distal changes in microbial communities and immune responses from the site of original perturbation. Intestinal perturbation by antibiotic treatment can shift the pulmonary immune response, and pulmonary perturbations can lead to shifts in intestinal microbiota. However, until recently, microbiome studies during infection focused on the influence of intestinal dysbiosis on pulmonary infections through the gut–lung axis as opposed to dysbiosis directly within the lung itself.

### 3.1. Influence of The Gut–Lung Axis during Viral and Bacterial Infections

The gut–lung axis refers to the direct effect that intestinal dysbiosis has on modulating immunological responses of pulmonary infections. This phenomenon has been observed in a plethora of bacterial infections ranging from *P. aeruginosa*, *S. pneumoniae*, and *M. tuberculosis* (TB) or viral infections like influenza A virusand RSV [12,15,16,17,18,19]. Although TB is caused by a bacterial pathogen, TB and *Coccidioides* infection have similar immunological responses. Both infections primarily elicit a Th1 and Th17 CD4+ T cell pro-inflammatory response and promote granuloma formation, a cluster of white blood cells that surround the infectious agent (see granuloma review [20]). Broad spectrum antibiotic treatment increases susceptibility to *M. tuberculosis* and modulates inflammatory responses in the lung of a TB infection mouse model [19]. Long non-coding RNA-chorionic gonadotropin subunit beta (lncRNA-CGB), directly controlled by *Bacteroides fragilis*, is downregulated by induced dysbiosis of gut microbiota and enhanced by oral administration of *B. fragilis* [19]. Enhanced lncRNA-CGB expression correlates with anti-TB immunity conferring immune protection [19]. In non-TB mycobacterial pulmonary disease (NTM-PD) patient and mouse models, metabolic serum L-arginine metabolite levels are significantly decreased relative to healthy or non-infected controls [21]. Oral administration of L-arginine enhances expansion of IFN- *γ*-producing T cells and shifts towards microbicidal macrophage responses in the lungs of NTM-PD mice [21]. Furthermore, fecal transplant from L-arginine-treated mice into NTM-infected mice increases host defense in the lungs and enriches gut *Bifidobacterium* species [21]. Thus, oral administration of *Bifidobacterium pseudolongum* enhances pulmonary immune defense in NTM infection [21]. In TB and NTM-PD infections, direct and indirect gut microbiome manipulation by different mechanisms results in distal effects on pulmonary immunity. Depleting the integrity and diversity of the microbiota has negative implications on host immune responses. Alternatively, reconstitution of compromised resident bacteria results in progressive priming of a productive and effective immune response against these infections [19,21]. However, there are also instances in which the host benefits from gut dysbiosis during an infection or the infection has a negligible impact on the host microbiome.

Respiratory influenza virus (PR 8) infection induces Th17 responses within the small intestine, altering intestinal microbiota composition and promoting intestinal injury [22]. Intestinal injury is not caused by direct intestinal infection, but rather by the increased Th17 cells and IFN- *γ* production [22]. Intestinal microbiota depletion by antibiotic treatment reverses intestinal injury by reducing local intestinal Th17 responses despite ongoing immune-mediated lung injury post-infection [22]. Neutralizing anti-IL-17A during PR 8 infection reduces intestinal injury, suggesting that PR 8 infection influences intestinal microbiota composition and promotes Th17 cell mediated intestinal injury [22]. Although *Coccidioides* infection is not correlated with intestinal injury that results in diarrhea, it is important to keep in mind that antibiotic use is not exclusively deleterious to pulmonary infections and in some situations it can be beneficial. Coinfections are common and may be a time in which antibiotic use is necessary and beneficial to the host. Negligible changes in the host microbiota are also observed in patients infected experimentally with RSV (Memphis 37); patients maintain normal bacterial load, alpha diversity, and species turnover in the upper respiratory tract microbiome [23]. This is in contrast to observations in children where RSV alters microbiome diversity, perhaps due to the more established immune system and microbial composition in adults, limited sample numbers, or microbial sample collection methods between these experiments [23]. A larger study group and sampling the lower respiratory system could yield different results; nonetheless, this study exemplifies that host microbiota change due to infection is dependent upon pathogen type and host responses. How gut and lung dysbiosis impact pulmonary and disseminated infections in Valley fever is unexplored and unknown. It cannot be assumed that *Coccidioides* infection alone would change the microbiota or that changes would be favorable or unfavorable for the host immune response. However, it is possible that perturbations to host microbiota caused solely by infection or coupled with antibiotic treatment would result in an altered pulmonary and intestinal microbiota and thus alter protective immune responses to this fungal pathogen. Antibiotic treatment allows access for opportunistic fungal infections to take hold (such as *Candida* and *Aspergillus* species) and warrants exploration for *Coccidioides* infection [24].

### 3.2. COVID-19 and Lung Microbiome

Viral and bacterial pathogens encompass many of the contracted respiratory infections; however, fungal pathogens are also a major cause of respiratory infections and are understudied. CDC estimates 7199 deaths from fungal disease in 2021 [25]. In 2019, there were 20,003 Valley fever infections mostly from Arizona and California with approximately 200 deaths each year [26]. Unfortunately, these statistics have likely been further exacerbated by challenges associated with the recent COVID-19 pandemic. Similarities in disease symptomology have contributed to delays in accurate diagnosis and delayed antifungal treatment, putting patients at higher risk for developing disseminated disease or even death [27]. Distinguishing between these infections was not the only challenge that the COVID-19 pandemic posed. COVID-19 showed the potential to stunt the host immune system, resulting in several invasive fungal infections that complicated the course of COVID-19 and increased mortality. Among these invasive fungal infections are *Pneumocytstis jirovecii* pneumonia, cryptococcosis, histoplasmosis, pulmonary *Fusarium* infection, *Scedosporium*, and *Coccidioides* infection [28]. Immunosuppressive medications or diseases increase coccidioidomycosis risk, and, in a case study, two patients with COVID-19 who required medication such as dexamethasone or glucocorticoid had a higher associated risk for *Coccidioides* reactivation [29]. The lymphopenia-mediated immune dysregulation associated with COVID-19 may also lower the immunity of hosts with *Coccidioides* infection [29]. These issues exclusively account for the direct immunological repercussions of a co-infection; however, COVID-19 has shed light on an underexplored field of infection-induced lung microbial dysbiosis. The lung microbiome has the ability to activate the innate and adaptive immune system; however, its influence on disease has long been neglected. A study comparing the lung microbial composition of bronchoalveolar lavage (BAL) fluid in COVID-19, community-acquired pneumonia, and healthy control patients found that COVID-19 and community-acquired pneumonia patients have more pathogen-enriched microbiotas than healthy controls [30]. Another study evaluating COVID-19 patient post-mortem lung biopsies revealed a mix of bacterial and fungal infections associated with fatal COVID-19 [31]. Many fungal pathogens, such as *Candida albicans*, are opportunistic, only causing pathology during immune “distraction” or dysfunction which might be aided by microbiota dysbiosis. This could also be true for *Coccidioides* infection. Risk groups most susceptible, in general, to developing chronic infection encompass patients with autoimmune diseases; thus, antibiotic-induced microbial dysbiosis may provide this niche for *Coccidioides* establishment and pathology.

### 3.3. Changes to Lung Microbiota Induced by Infection: Bacterial, Viral, Fungal, and Parasitic

Infection alone accounts for perturbation-induced changes to the host microbiome. Aside from COVID-19-induced lung specific dysbiosis, dysbiosis of the lung microbiota is observed during pulmonary TB and HIV infections. The first study to analyze the microbial composition in the lower respiratory tract of patients with TB revealed that the microbial composition of infected patients is more diverse than that of healthy participants [32]. Actinobacteria phyla is overrepresented in TB patients, while Bacteroidetes and Fusobacteria phyla are underrepresented [32]. Genera *Phenylobacterium, Stenotrophomonas, Cupriavidus, Caulobacter, Pseudomonas, Thermus,* and *Sphingomonas* are unique to and widely distributed among pulmonary TB patients [32]. Some of these abnormal genera, such as *Phenylobacterium* and *Sphingomonas*, are associated with infectious diseases, suggesting that pulmonary TB creates a niche favorable for foreign and potentially pathogenic bacterial growth [32]. This pathogen favorable niche may be as a result of lesions in the lung tissue caused by the pathogen or host immune responses to infection that, in turn, allow a new niche to invade and colonize [32]. The host must effectively distinguish between symbiotic and foreign threats to elicit an appropriate immune response. While the host deciphers between resident or invader, the initial immune response may kill some symbiotic bacteria, allowing the invaders to contribute to and exacerbate pathology caused by the infection itself. Interleukin-36*γ* has been identified as a global discriminator of pathogenic and resident microbes [33]. Interleukin-36*γ* is upregulated in response to pathogen-associated molecular patterns present on all microorganisms but is activated through proteolytic cleavage by extracellular proteases as a result of cellular damage [33]. However, other non-specific inflammatory cytokines may also be released, initiating inflammation and inducing epithelial damage. For instance, in vitro interactions between multiple fungi and epithelial cells reveal morphological changes in epithelial cells such as rounding and detachment, along with cell injury of the epithelial cell plasma membrane [34]. Thus, epithelial damage may be negative for the host but positive for opportunistic invaders and pathogens. The first lung microbiome TB study revealed differences in infected and healthy individuals. Studying the lung microbiome during infection gained traction and further investigations were performed. Pre- and post-infection microbial interaction networks of macaques revealed the reorganization of microbial communities within the lung [35]. Using a model that infers conditional independence between operational taxonomic units, a notable decrease was observed in the number of microbial interactions post-TB infection [35]. Some taxa lost all connections, indicating independence under TB infection conditions [35]. Other taxa gained connections, some with positive and some with negative correlations, suggesting that TB competes with specific taxa and can either subdue or create a symbiotic relationship with other taxa [35]. Microbial interaction networks during TB are coupled with variable microbial changes, with some macaques experiencing large microbial shifts and others holding stable communities [35]. This varied microbial diversity could contribute to the spectrum of disease progression observed in TB. No single species was identified correlating with TB disease outcome; however, HIV-infected human subjects have a significant abundance of *Tropheryma whipplei* in their BAL fluid [36]. Treatment with anti-retroviral therapy reduces *T. whipplei* abundance in the lung [36]. These studies examined the airway microbial shifts that occur as a result of bacterial and viral infection and the detrimental implications of these shifts.

Experimental infection studies have been predominantly focused on bacterial and viral infections; however, parasitic and fungal infections also cause dysbiosis. *Paragonimus proliferus* infection changes the composition of lung microbiota and increases the prevalence of potentially pathogenic microbes [37]. Alongside bacterial and viral infections, other parasitic infections by *T. gondii* and *E. histolytica* also cause changes to the gut microbiota and increase susceptibility through modulation of the immune response [38,39]. Most studies on fungal infection-induced dysbiosis have focused on the gut, not the lung, despite these being respiratory infections. Intestinal microbiota diversity is significantly altered by respiratory infection with *Pneumocystis murina* [40]. Absence of microbiota in germ-free mice increases susceptibility to *Cryptococcus gattii* as depicted by reduced survival, higher fungal burden in the lungs and brain, and reduced proinflammatory cytokines [41]. Restoring gut microbiota with fecal transplants from conventional mice or lipopolysaccharide (bacterial surface) administration increases survival and levels of inflammatory mediators [41]. Although these studies demonstrate infection-induced gut dysbiosis, changes to lung microbiota were neglected until recently, when studies on invasive pulmonary aspergillosis revealed that the lung microbiota is altered [42]. Invasive pulmonary aspergillosis patients display a decreased alpha diversity and increased or decreased proportions of distinct genera [42]. This study explored the association between lung bacterial composition and lung immunity using distance matrices and found a gradient in the number of neutrophils [42]. This indicates a shift in the overall composition of the lung microbiome according to neutrophil count [42]. Neutrophil counts positively correlate with bacterial reads and increase with the abundance of rare and understudied bacterial taxa [42]. However, most studies do not examine the relationship between lung microbial composition and specific host immunological responses. Thus, these infections and the pandemic highlight the need for studying the lung microbiome interaction with immunological responses during infection.

## 4. Productive Immune Responses against *Coccidioides*

The immune system is divided into two branches: the innate (primary, general) immune system and the adaptive (secondary, specialized) immune system. Neutrophils, macrophages, and dendritic cells are part of the innate immune system and influence the clearance of *Coccidioides* (see [43] for detailed review). High neutrophil presence is associated with chronic *Coccidioides* infection [44]. This could potentially be due to the ability of neutrophils to re-enter circulation and deposit the pathogen at sites distal to site of infection [44]. Macrophages also engulf fungi; however, in vitro macrophages phagocytose *Coccidioides* arthroconidia poorly and, although we would expect a proinflammatory phenotype, macrophages do not polarize into proinflammatory (M1) or anti-inflammatory (M2) [45]. *Coccidioides* spherules are also poorly phagocytosed by macrophages, perhaps due to the large size of the spherules allowing for prolonged fungal infection [46]. Dendritic cells are professional antigen-presenting cells and are responsible for activating naïve T lymphocyte. Dendritic cells from healthy patients pulsed with *Coccidioides* spherule lysate induce antigen-specific T cell activation [47]. A specific and productive adaptive immune response is crucial for clearance of infection. As the first line of defense, the innate immune system primes an effective adaptive immune response. Thus, improper innate immune responses are detrimental in shaping host immunity.

T lymphocytes are a part of the adaptive immune system and play a major role in *Coccidioides* infection. Activated CD4+ T cells differentiate into T helper (Th) subsets (Th1, Th2, or Th17) that aid in combating infections and protecting the host. Th1 and Th17 cells are required for *Coccidioides* clearance [48]. IL-12 produced by macrophages and dendritic cells promotes the differentiation of IFN-*γ* producing Th1 cells [49]. When recombinant IL-12 was administered to *Coccidioides* susceptible BALB/c mice, shifts from Th2 to Th1 associated cytokines coinciding with reduced lung, liver, and splenic fungal burden were observed [50]. Th1 and Th17 cells are also required for memory responses to protective *Coccidioides* vaccination [51]. As described in non-TB mycobacterial pulmonary disease studies above, the host microbiota regulates IFN- *γ* producing cells via metabolite production. Metabolite-producing host microbiota depleted by antibiotic use during early *Coccidioides* infection may skew the immune response away from IFN- *γ*/Th1 responses, altering protective immunity in unknown ways.

## 5. Changes to Lung Microbiota Induced by Antibiotic Treatment Alters Immune Response to Infection

As discussed previously, infection alone causes changes to the host microbiota and certain genera are associated with severe disease. Antibiotic treatment is also known to cause changes to the lung and gut microbial repertoire. Erythromycin is positively correlated with Fusobacteria and negatively correlated with Bacillaceae [52]. Clarithromycin, on the other hand, is positively correlated with Synergistetes and Verrucomicrobia phylum [52]. Atopobiaceae and Fusobacteriaceae are positively correlated with all antibiotics in the lungs [52]. However, these microbial associations may be altered by the combination of antibiotics or antibiotic treatment plus infection in a way that renders the host more susceptible to infection due to altered immune responses. The negative effect of antibiotic perturbation on respiratory immune responses to infection is observed in lymphocytic choriomeningitis virus, influenza A virus, RSV, and Sendai virus [11,53,54,55] (Table 1). RSV challenge elevates inflammatory cells in the bronchioalveolar lavage fluid of mice, but streptomycin treatment further exacerbates RSV-induced inflammatory cell infiltration into the lung [11]. Streptomycin treatment shifts the immune response from an effective anti-RSV Th2 to a Th1/Th17 response that likely induces high inflammation [11]. In influenza A virus infection, treatment with vancomycin, neomycin, metronidazole, and ampicillin cocktail reduces influenza-specific antibody titers and CD4 T cell responses while increasing lung viral titers [53]. This indicates that changes in the microbiota have a direct effect on adaptive immune responses to influenza A virus [53]. Productive antiviral adaptive immune responses are restored by one inoculation of LPS intranasally or intrarectally [53]. Thus, even though the intestinal microbiota density is much greater than that of the lung, lungs are also inhabited by microbial species that can be impacted by antibiotics and influence the respiratory immune response to infections. However, the lung microbiota composition and its influence on the immune response were not considered in these studies. The study highlights that antibiotics’ influence on adaptive immune responses during respiratory infections varies depending on the infectious agent or route of infection [53]. These data further suggest that an intact microbiota is not a general requirement for effective immunity during infections and several factors may contribute to the disease outcome.

**Figure 1:**
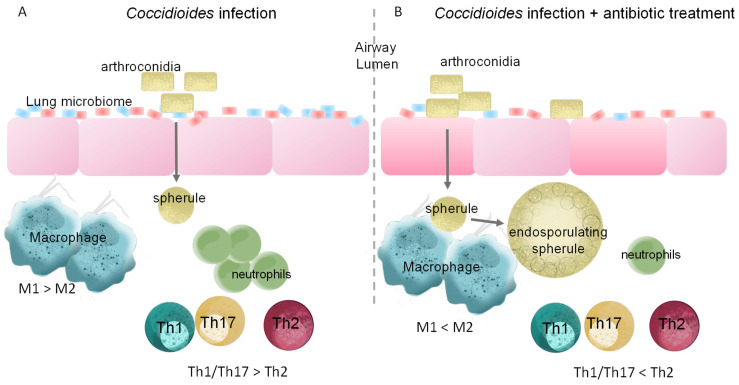
Speculative lung commensal microbiota, immune cell, and *Coccidioides* interactions based on other infection studies. (**A**) Infection alters resident lung commensals (blue = symbiotic bacteria; red = pathogenic bacteria), altering the diversity and increasing pathogenic bacteria. Productive immune response against *Coccidioides* with an inflammatory macrophage (M1) response over anti-inflammatory macrophage (M2) response, predominant CD4 Th1 and Th17, and robust neutrophil response. (**B**) Antibiotic depleted microbiome allows a niche for facilitated *Coccidioides* entry into the lung, while antibiotics also alter the host immune response possibly shaping an ineffective immune response to *Coccidioides*.

Two studies investigating diagnosis and treatment of coccidioidomycosis identified that 70% of Valley fever patients are treated with one or more rounds of antibiotics prior to accurate diagnosis [59,60]. Inaccurate diagnosis led 54% of patients to receive initial antifungal treatment at >4 weeks in one study and 79% of patients to receive initial antifungals a year after coccidioidomycosis diagnosis in another study [59,60]. Early or late antifungal treatment did not yield differences in clinical outcome or timing of IgG complement fixation titer decrease in *Coccidioides* infected inmates [59]. However, other studies have shown that early antifungal treatment decreases peak IgG complement fixation titers, dissemination, and chronic disease [59,61]. Regarding antibiotic treatment, Filipinos have the highest percentage of persons receiving antibiotics, despite sample size being relatively small [60]. Filipinos have a 140-fold higher risk for developing severe or disseminated disease; thus, this correlative data highlights the controversy surrounding the effects of delayed diagnosis and improper antibiotic treatment on the development of chronic *Coccidioides* [60]. The most common antibiotics used in these studies were azithromycin, ceftriaxone, doxycycline, ciprofloxacin, and vancomycin, or trimethoprim-sulfamethoxazole, erythromycin, and doxycycline, respectively [59,60]. Among these, vancomycin is considered a non-absorbable antibiotic, meaning that it is poorly absorbed systemically and acts locally in the gut. However, oral administration of vancomycin alters the murine lung microbiome and pulmonary immune responses [62]. These changes may be attributed to translocation of bacteria or bacterial metabolites from the gut to the lung via the gut–lung axis; however, further studies are necessary to clarify the route and mechanism of action [62]. Most antibiotics have good penetration into the lung [63]. Absorbable antibiotics can cause direct distal changes to the lung microbiota. Additionally, treatment with different antibiotics results in distinct pulmonary outcomes in allergic airway inflammation [64]. Amoxicillin- and trimethoprim/sulfamethoxazole-treated mice display increased airway hyperresponsiveness, reduced lung alveolar volume, and increased levels of proinflammatory cytokines in the bronchioalveolar lavage fluid, indicative of increased inflammation [64]. Metronidazole treatment, on the other hand, is associated with increased antigen-specific IgA in mouse serum associated with decreased asthma prevalence [64]. Although pathogen mediated responses may differ from allergic inflammation, these findings highlight that antibacterial agent choice may have different impacts and potential disease outcomes in coccidioidomycosis [64]. Nonetheless, since Valley fever patients are often treated with antibiotics pre- and post-coccidioidomycosis diagnosis, these antibiotics likely cause perturbations on the host microbiota and immune response, making it worthwhile to explore these effects during coccidioidomycosis (Figure 1). Additionally, a soil *Bacillus subtilis-*like species displaying antifungal activity against *Coccidioides* growth has been identified [65]. Whether host commensal bacteria also inhibit *Coccidioides* by direct or indirect mechanisms is unknown. However, we have observed intestinal and tracheal microbiomes inhibit the growth of *Coccidioides* in vitro (Tejeda-Garibay, S., Hoyer, K.K., unpublished data). Furthermore, it is unknown how antibiotic treatment resulting from misdiagnosis further affects the interrelationship between the host lung microbiome and the invading fungal pathogen. Future studies are needed to inform clinicians on the diagnosis and treatment of coccidioidomycosis and the potential consequences of prescribing antibiotics during a fungal infection.

## 6. Conclusions and Perspectives

### 6.1. Microbiota and Immune Features for Diagnostic Use

In lung diseases such as cystic fibrosis, chronic obstructive pulmonary disease, COVID-19 and TB, changes in microbiota diversity correlate to disease severity [13,30,66,67]. In COVID-19, immune parameters and respiratory tract composition correlate to disease severity and are proposed as diagnostic tools [13]. Severe COVID-19 disease correlates positively with lower abundance of *Haemophilus*, *Actinomyces*, and *Neisseria* genus compared to normal oropharyngeal microbiome [13]. Correlation of the oropharyngeal microbiome with the systemic immune response showed lower lymphocyte-to-neutrophil ratio associated with lower diversity, and composition of oropharyngeal microbiome inversely correlated with disease severity [13]. Moreover, machine learning algorithms reveal that features of the microbiome at early sampling points discriminate the level of COVID-19 severity and could potentially be used as a diagnostic tool [13]. These parameters inform the state of infection and clinical management approaches. In TB, a heatmap based on the top 100 species in BAL fluid of an untreated pulmonary TB group, a healthy control group, and a lung cancer patient group comprehensively display clear differences in untreated TB patients versus healthy TB and lung cancer patients [30]. In COPD, high severity is positively correlated with a decrease in bronchial microbiome diversity [66]. There is partial loss of the resident microbiota that is replaced by a microbiota that includes potentially pathogenic microorganisms (*H. influenzae*, *P. aeruginosa*, *S. pneumoniae*, *M. catarrhalis*) [66]. The same phenomenon is observed in cystic fibrosis with the recognized cystic fibrosis pathogens (*P. aeruginosa*, *S. aureus*, *S. maltophilia,* and *B. cepacia)* becoming prevalent as lung function decreases [67]. Overall, findings in each pulmonary disease suggest that surveying the microbiota composition can serve as a predictive factor of disease severity and could inform treatment options.

### 6.2. Probiotics and Bacterial Products as a Therapeutic Option

Studies on bacterial, viral, and fungal pathogens have observed altered immune responses because of antibiotic-induced dysbiosis. Probiotics and prebiotics rescue the effects of dysbiosis. Probiotics are live microorganisms and prebiotics are typically carbohydrates broken down that serve as a source of food for bacteria. Bacteria then release metabolites (short chain fatty acids) that affect immune responses. The most common probiotics used are species within the *Lactobacillus*, *Bifidiobacterium*, and *Bacillus* genus. Independent of the type of infection, use of probiotics tends towards increasing survival, decreasing pathogen load, or modulating immune responses towards a productive immune response [68,69,70,71,72,73]. Prebiotics such as inulin, polysaccharide, and oligosaccharide have similar effects on infection [69]. The first step in coccidioidomycosis studies should be to identify the effects of infection and antibiotic treatment on the host lung and gut microbiota. Once established, observed alterations to the host microbiota could be defined and further studied. Beneficial bacteria and their metabolites could be used assessed for therapeutic use to correct for dysbiosis and prime for productive immune responses.

### 6.3. Future Directions for Coccidioidomycosis Research

Literature exists on the effects that microbial dysbiosis has on viral and bacterial infections; however, it is lacking in the fungal infection field and is non-existent for coccidioidomycosis infection. Coccidioidomycosis is a respiratory infection like many of the viral and bacterial infections discussed in this review. As outlined, respiratory disease progression and outcome are highly influenced by dysbiosis and thus similar concerns regarding the effects of dysbiosis on coccidioidomycosis are warranted. The added layer of improper antibiotic treatment driven by misdiagnosis further exacerbates this concern regarding the effects of an altered microbiome on infection. *Coccidioides* research and clinical care will benefit from broad global microbial and immune knowledge and specific symbiotic/antagonistic species identification during infection. Such findings will inform implementation of preventative, diagnostic, and therapeutic tools.

## Figures and Tables

**Table 1:** Bacterial and viral studies indicating the effects of antibiotic use on the microbiota, immune response, and infection outcome.

Infection	Antibiotic(s)	Δ Intestinal Microbiota	Δ Respiratory Microbiota	Δ Immune	Infection Outcome	Reference
Respiratory Syncytial Virus	Streptomycin sulfate	↓ *Lactobacillus*, *Clostridium_XlVa*, *Alistipes* ↑ *Bacteroides*	N/A	↑ eosinophils, lymphocytes, neutrophils (inflammatory cells) ↑ IFN- *γ*, IL-17A ↓ IL-10, IL-3, IL-4, IL-5	No effect on viral load Enhanced pulmonary inflammation Dysregulated immune response	[11]
Influenza A virus	Vancomycin (V), Neomycin (N), Metronidazole(M), or ampicillin(A)	↓ bacterial load and dominated by *Sphingomonas* species(N) ↓ Gram+ bacteria(A) ↑ *Enterobacter* species	Dominated by *Lactobacillus* species (V & M) ↓ Gram+ bacteria (A) ↑ *Enterobacter* species	↓ CD 8 T cell responses (neomycin)	↑ viral load (V, N, M, A) ↓ pro-IL-1β, pro-IL-18, NLRP3 (V, N, M, A) ↓ mLN total #, proliferation, differentiation, activation and migration of DCs (V, N, M, A) Impaired immune responses (N)	[53]
Sendai virus	Streptomycin	↓ alpha diversity ↑ *Bacillales*	No significant differences	↑ IL-6, IFN- *γ*, CCL2, CCL11 ↑ %NK1.1-expressing lymphocytes ↓ Foxp3+ Tregs	↑ mortality	[54]
Lymphocytic Choriomeningitis Virus	Ampicillin, Gentamicin, Metronidazole, Neomycin, Vancomycin, and sucralose	↓ commensal bacteria	N/A	↓ CD8+ T cell responses ↓ IgG antibody titers ↑ T cell exhaustion (PD-1, 2B4 CD160, LAG-3) ↓ innate antiviral immune responses	↑ viral titer in kidneys Delayed viral clearance	[55]
*Streptococcus pneumoniae*	Ampicillin, Neomycin, Metronidazole, and Vancomycin	↓ microbial diversity	N/A	↑ IL-1β, IL-6, and CXCL1 ↓ TNF-α and IL-10	↑ mortality rate ↑ bacterial load ↑ lung neutrophil influx ↑ tissue inflammation, liver damage, and hepatic injury ↓ alveolar macrophage phagocytosis capacity	[15]
*Klebsiella pneumoniae*	Ampicillin, Neomycin sulfate, Metronidazole, and Vancomycin	N/A	N/A	↓ IL-6, TNF-α ↓ bacterial killing by alveolar macrophages ↓ H_2_O_2_	↑ bacterial burden	[56]
*Escherichia coli*	Ampicillin, Vancomycin, Neomycin sulfate, and Metronidazole	Not detectable (no sequencing)	N/A	↓ myeloperoxidase activity ↓ TNF-α production ↓ alveolar macrophage bacterial killing ↑ IL-6 and IL-1β ↓ NF-kβ DNA-binding activity in intestinal mucosa and lung ↓ TLR4, TNF-α, KC, ICAM, and CXCR2 expression in intestinal mucosa ↑ IL-1β, KC, and MIP-2 in the lung	↑ bacterial burden ↑ bacteria in blood ↑ mortality ↑ interstitial edema, septal edema, and alveolar edema in lung	[57,58]
*Pseudomonas aeruginosa*	Vancomycin-colistin	↓ diversity ↓ *Muribaculaceae*, *Prevotellaceae*, and *Lachnospiraceae* ↑ *Burkholderiaceae*, *Clostridiales*, *Lactobacillaceae*	No change	↓ macrophages, cDC2, inflammatory monocytes, neutrophils, iNKT cells ↓ Flt3-ligand	↑ *P. aeruginosa* load in lung and spleen ↑ lung injury ↓ survival	[12]

Abbreviations: N/A = not evaluated; DC = dendritic cell; mLN = mesenteric lymph node; ↑ = increase; ↓ = decrease; *γ =* gamma.

## Data Availability

No new data were created.
